# Self-Navigated 3D Acoustic Tweezers in Complex Media Based on Time Reversal

**DOI:** 10.34133/2021/9781394

**Published:** 2021-01-04

**Authors:** Ye Yang, Teng Ma, Sinan Li, Qi Zhang, Jiqing Huang, Yifei Liu, Jianwei Zhuang, Yongchuan Li, Xuemin Du, Lili Niu, Yang Xiao, Congzhi Wang, Feiyan Cai, Hairong Zheng

**Affiliations:** ^1^Paul C. Lauterbur Research Center for Biomedical Imaging, Key Laboratory for Magnetic Resonance and Multimodality Imaging of Guangdong Province, Shenzhen Key Laboratory of Ultrasound Imaging and Therapy, Shenzhen Institutes of Advanced Technology, Chinese Academy of Sciences, Shenzhen 518055, China; ^2^Shenzhen College of Advanced Technology, University of the Chinese Academy of Sciences, Beijing 100049, China; ^3^Verasonics, Inc., WA 98034, USA

## Abstract

Acoustic tweezers have great application prospects because they allow noncontact and noninvasive manipulation of microparticles in a wide range of media. However, the nontransparency and heterogeneity of media in practical applications complicate particle trapping and manipulation. In this study, we designed a 1.04 MHz 256-element 2D matrix array for 3D acoustic tweezers to guide and monitor the entire process using real-time 3D ultrasonic images, thereby enabling acoustic manipulation in nontransparent media. Furthermore, we successfully performed dynamic 3D manipulations on multiple microparticles using multifoci and vortex traps. We achieved 3D particle manipulation in heterogeneous media (through resin baffle and *ex vivo* macaque and human skulls) by introducing a method based on the time reversal principle to correct the phase and amplitude distortions of the acoustic waves. Our results suggest cutting-edge applications of acoustic tweezers such as acoustical drug delivery, controlled micromachine transfer, and precise treatment.

## 1. Introduction

The trapping or transportation of micro- or nanoparticles is critical when attempting to catalyse a desired macroscopic effect via the manipulation of substances in the microscopic world. Optical tweezers [1, 2] are widely used tools for particle manipulation, and this has led to the development of a few cutting-edge applications in the field of biology and physics, such as evaluating the shear modulus of the human erythrocyte membrane [3], measuring the DNA spring constant [4], studying the dynamic characteristics of the kinesin movement [5, 6], measuring the instantaneous velocity of a Brownian particle [7], and trapping cold atoms [8]. Despite these noteworthy applications, optical tweezers suffer from a fundamental constraint in that light cannot traverse nor focus inside optically turbid media such as the human body. Therefore, optical tweezers can only be applied to a limited range of applications.

Acoustic tweezers recently emerged as an alternative tool for manipulating particles in a much broader range of media. Acoustic waves exert acoustic radiation forces on objects because of the momentum transfer that arises from sound scattering or attenuation. Acoustic radiation force has been demonstrated to manipulate several types of microparticles [9–11] in a noninvasive or noncontact manner. Because the velocity of acoustic waves is significantly smaller than that of light, the acoustic radiation force is typically five orders of magnitude greater than optical radiation force for a given input power; this enables the manipulation of more massive particles without increasing the risk of tissue ablation [12, 13]. Moreover, acoustic waves can propagate in transparent as well as optically turbid media irrespective of their optical refractive index, which considerably broadens their range of applications in comparison to that of optical tweezers (for example, *in vivo* particle manipulation [14, 15]). Currently, the manipulation of microbubbles is a typical application direction of acoustic tweezers, such as acoustic trapping and releasing of microbubbles in complex environments [16], transporting microbubbles using surface acoustic waves [17], and patterning of microbubbles [18].

Acoustic tweezers can be broadly classified into two categories [19], i.e., collective tweezers, which manipulate multiple particles undergoing the same behaviour [20], and selective tweezers, which create various traps that independently manipulate a subgroup of particles undergoing different behaviours [21–23]. Recently, artificial acoustic structures have been applied for complex beam generation and acoustic tweezers, such as waveguiding obtained by a superhydrophobic acoustic metasurface [24], generation of acoustic acceleration beams via flexible active surfaces [25], superoscillation wave packets using a metalens [26], realization of sound deceleration with helical-structured acoustic metamaterials [27], and fine acoustic manipulation via lossy metamaterials [28]. Although the acoustic fields obtained by artificial structures have a high spatial resolution, they are generally fixed and lack flexible dynamic characteristics. However, both collective and selective tweezers require a dynamic update of the acoustic wave fields. A phased array is preferred over single-element transducers because the amplitude and phase of the emitted signal from each element can be adjusted independently, thereby enabling arbitrary acoustic wave fields to be synthesized, updated, and moved in real time; this helps in enriching the types and modes of particle manipulation. For example, a controllable Bessel beam has been used for the precise manipulation of the mixture and stirring liquid microparticles [29]. Further, acoustic vortices have been reported to trap Mie particles in the air [30]. Meanwhile, the concept of holography has been applied to acoustic tweezers to levitate objects with twin, vortex, and bottle traps using single-sided arrays [21] and to dynamically manipulate multiple particles simultaneously and independently in a 3D space using a double-sided arrangement of two opposed arrays in the air [31]. Recently, particle manipulation which combines phased array and artificial acoustic structures has been proposed to integrate the advantages of the two methods [32].

While the 3D acoustic tweezers showed effective performance in the air [21, 23, 31, 33], for several reasons, it was rarely replicated in water, wherein the acoustic impedance is close to that of the human tissue. Currently, acoustic tweezers in water are mainly implemented using standing surface acoustic waves (SSAWs) [34, 35], which are commonly generated by interdigitated transducers. The SSAWs are typically operated at a considerably higher frequency range (tens to hundreds of megahertz [36]) to separate and manipulate tiny particles and cells. However, SSAWs encounter challenges such as the need for additional microfluidic systems, the limitations of the acoustic field patterns, and the small space for manipulating particles, especially in the vertical direction due to sound waves travel along the surface [37]. Acoustic streaming, which is generated by boundary-layer effects, was also used to manipulate microparticles in water environment, such as dynamic particle concentration and translation in an open fluid well [38] and acoustofluidic salivary exosome isolation [39]. To manipulate particles on a large scale in a 3D space, bulk acoustic waves generated by a matrix array need to be applied, which provide a critical basis for developing 3D acoustic tweezers in water. Sound attenuation drastically reduces in water; therefore, the operable frequency of the acoustic tweezers can be increased significantly to improve the precision of manipulation. This results in a considerably finer element pitch required for the matrix array to match the same trapping beam steerability as that of the airborne acoustic tweezers. Currently, the challenge exists in manufacturing an appropriate matrix array and a driving electronic system.

There are several more challenges for 3D acoustic tweezers *in vivo*. On the one hand, optical imaging cannot be applied to deep tissues, which makes it challenging to track the particle locations inside the human body—a prerequisite for acoustic tweezer beamforming. On the other hand, *in vivo* structures are complex and therefore require acoustic trapping beams carefully designed considering possible phase abbreviations or distortions caused by heterogeneous tissue structures such as human bones and skulls.

**Figure 1:**
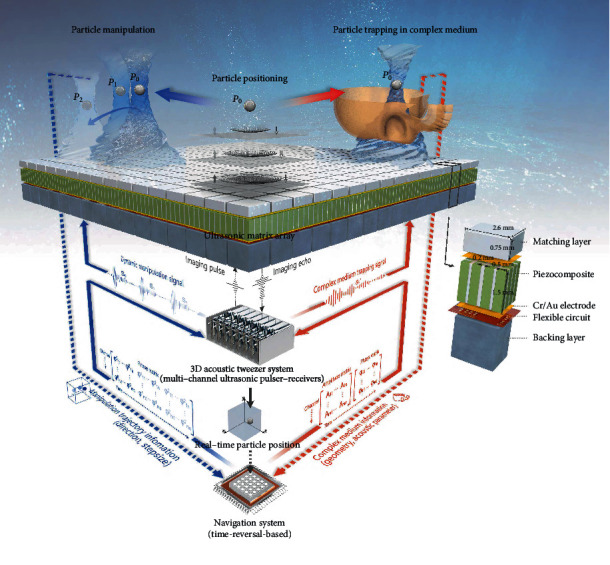
Schematic of self-navigated 3D acoustic tweezers in a complex medium based on the time reversal principle. The 3D acoustic tweezers were realized in water based on a 256-element ultrasonic matrix array. The 3D acoustic tweezer system is a multichannel ultrasonic pulser-receiver, which can generate various acoustic fields in real time; thus, it is feasible to manipulate the particles. Further, this system can also transmit imaging pulses and generate 3D ultrasonic images using imaging echo signals (black loops in the middle) to track the particle position (*P*_0_). The navigation system refers to a high-performance workstation capable of running a simulation program, which dictates acoustic tweezer beamforming. The red loops indicate the process of time-reversal based 3D acoustic tweezers in heterogeneous media (such as manipulating the particle in a 3D trajectory through an *ex vivo* human skull), wherein complex medium information (red dashed line) is imported to the navigation system for calculation. The blue loops indicate the process of obtaining self-navigated 3D acoustic tweezers in water with the 3D manipulation trajectory (from *P*_0_ through *P*_1_ to *P*_2_, blue dashed line) as input.

In this study, we aim to tackle these challenges by developing a self-navigated, time-reversal-based 3D acoustic tweezer system. The system consists of a 1.04 MHz, 256-element 2D matrix array; programmable 256-channel pulser-receiver system; and navigation system that dictates acoustic tweezer beamforming (Figure 1). The matrix array first transmitted imaging pulses. A volumetric ultrasound image was reconstructed with the received echo signals to indicate the current locations of the particles; these locations were then fed into the navigation system in real time to formulate the trapping beams to seize the particles (Figure 1, black loops in the middle). The trapping beams were formed based on the time reversal principle. For a complex medium, such as when a skull was present in the acoustic travel path, the navigation system first emulated received signals as if originated from the desired focal spots. It then controlled the ultrasound electronics to transmit the emulated waveforms from each element in the time-reversed order; thus, the trapping beams were focused at the desired locations by assuming the reciprocity of the sound travel paths (Figure 1, red loops). For homogeneous media such as water, beamforming is considerably simplified because the acoustic travel time from each element to the focal spot is known. The navigation system, in this case, simply calculated the appropriate phase delays from each element and passed them to the ultrasound transmission electronics (Figure 1, blue loops). Once the particles had been trapped at the initial location, the navigation system started to dictate the ultrasound electronics to manipulate the particles. The navigation system divided the desired path into a finite number of sections, and within each section, trapping beams—similar to the aforementioned process—were formed based on the time reversal principle. Imaging pulses were interleaved between the trapping beams, and they reported the real-time locations of particles as feedback to the navigation system. Thus, real-time imaging enabled the visualization and monitoring of the entire process of particle manipulation.

## 2. Results

### 2.1. Self-Navigated 3D Acoustic Tweezers

**Figure 2:**
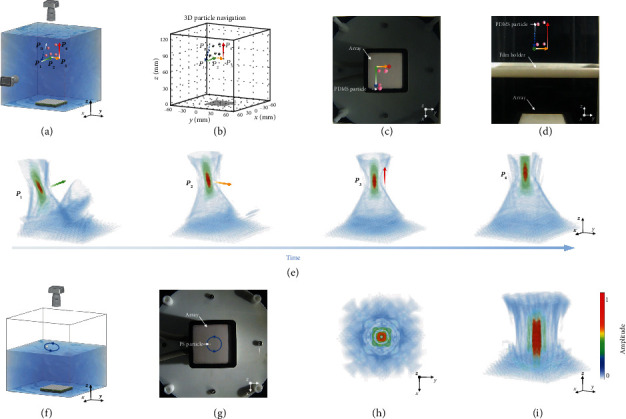
Self-navigated 3D acoustic tweezers. (a) Schematic of self-navigated 3D acoustic tweezers. The freely falling PDMS particle dropped from an arbitrary initial position was tracked by the 3D ultrasonic images at position *P*_0_ and the navigation system trapped the particle at *P*_1_. Then, the particle was transferred along a specified path (passing through *P*_2_ and *P*_3_) until it arrived at target position *P*_4_. (b) 3D ultrasonic images monitoring the particle manipulation path in 3D space. (c, d) Optical images monitoring the particle (dyed red) manipulation path in the top view (c) and side view (d). (e) Simulated 3D beam profiles at positions *P*_1_, *P*_2_, *P*_3_, and *P*_4_. (f, g) Schematic (f) and optical images (g) of manipulating a PS particle in a circular path (10 mm diameter) on the water surface. (h, i) Simulated 3D beam profiles of the vortex acoustic field from the top view (h) and side view (i). The self-navigated 3D acoustic tweezers are shown in Movie S1 and the manipulation of a single PS particle is shown in Movie S2. All scale bars are 10 mm.

In this section, we presented the results of manipulating two different types of particles—polydimethylsiloxane (PDMS) and polystyrene (PS)—in water using the self-navigated 3D acoustic tweezer system (Figures 2(a)–2(i)). Particles made of PDMS and PS are considered “soft” and “rigid,” respectively, as determined by their relative acoustic impedance to water.

As shown in Figure 2(a), a PDMS particle fell from an arbitrary location above the 2D matrix array within its footprint and sank freely in water. The matrix array first transmitted imaging pulses to track the particle location (*P*_0_ to *P*_1_). Once the particle landed at the desired depth (*P*_1_), the navigation system immediately switched the ultrasound electronics to the particle-trapping mode, thereby forming a focused wave field at *P*_1_ to trap the particle temporally. Meanwhile, the navigation system was loaded in a user-defined path from the trapping position (*P*_1_) to the target position (*P*_4_), and it subsequently dictated the ultrasound electronics to manipulate the particles to move along the path. After arriving at the target position (*P*_4_), the particle was trapped there to levitate until the next command was given. This result showed that unlike conventional acoustic manipulation, particles need not be artificially placed near the traps initially. The volumetric ultrasound images—acquired using the coherent plane-wave compounding (CPWC) [40]—clearly display the position of the particle in 3D (Figure 2(b)), thereby automating the ultrasonic electronics for acoustic tweezer beamforming. To cross-validate the ultrasonic navigation, we acquired the optical images shown in Figures 2(c) and 2(d)) and Movie S1. The simulated trapping beam profiles at the nodes of the path (*P*_1_ to *P*_4_) are shown in Figure 2(e). Further, a PS particle with a diameter of 0.5 mm was trapped by the vortex acoustic field and transferred in a circular path by changing the centre of the vortex (Figures 2(f) and 2(g)). The simulated acoustic wave fields of the vortex are shown in Figures 2(h) and 2(i).

### 2.2. Three-Dimensional Acoustic Tweezers for Multiple Particles

**Figure 3:**
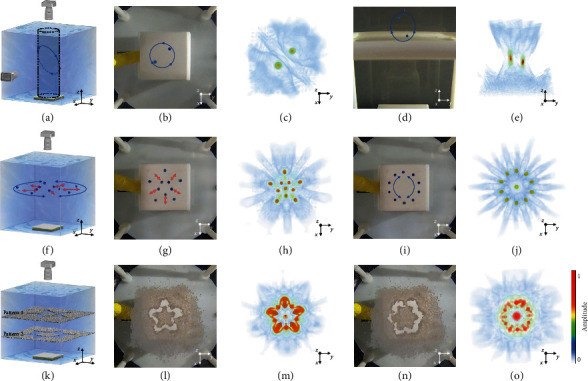
3D acoustic tweezers for multiple particles: (a) schematic of trapping two PDMS particles in 3D space simultaneously; (b–e) optical images (b, d) of two trapped particles (dyed blue) and corresponding simulated 3D beam profile (c, e) in the top and side view, respectively; (f) schematic of manipulating ten PDMS particles to transform from a circle to a pentagon and rotate in clockwise and counterclockwise directions; (g–j) optical images of ten trapped particles arranged into a pentagon (g) and a circle (i) in the top view and the corresponding simulated 3D beam profiles (h) and (j) respectively; (k) schematic of manipulating a large number of PS particles to display patterns of the pentagon and snowflake acoustic fields; (l–o) optical images of the pentagon pattern (l) and snowflake pattern (n) in the top view and the corresponding simulated 3D beam profiles (m) and (o), respectively. The acoustic manipulation processes for the two types of multiple particles are shown in Movie S2. All scale bars are 10 mm.

In this section, we showed the result of manipulating multiple particles in 3D space using the 2D matrix array. We started by manipulating two PDMS particles along an oblique elliptical path (Figure 3(a)); the particles were 1.5 mm in diameter. Figures 3(b) and 3(d) show snapshots of the particle movement projected in the *x*-*y* and *y*-*z* planes. Figures 3(c) and 3(e) are the simulated acoustic wave fields corresponding to Figures 3(b) and 3(d), respectively. We showed the collective manipulation of ten particles spinning around a circle (Figures 3(i) and 3(j)) and selective particle manipulation (Figures 3(f)–3(h)), wherein each of the ten particles was moved along the radial direction, transforming the circular pattern into a pattern of a plum blossom.

A large number of PS particles with approximate diameters of 400–800 *μ*m were laid flat on the film to display the acoustic field pattern. The radiation force exerted on the PS particles pushed the particles away from the areas with intense acoustic pressure, thereby displaying acoustic field patterns in blank areas (Figures 3(k)–3(o)). The acoustic manipulation of two types of particles is shown in Movie S2.

### 2.3. Time-Reversal-Based Acoustic Tweezers in Heterogeneous Media

**Figure 4:**
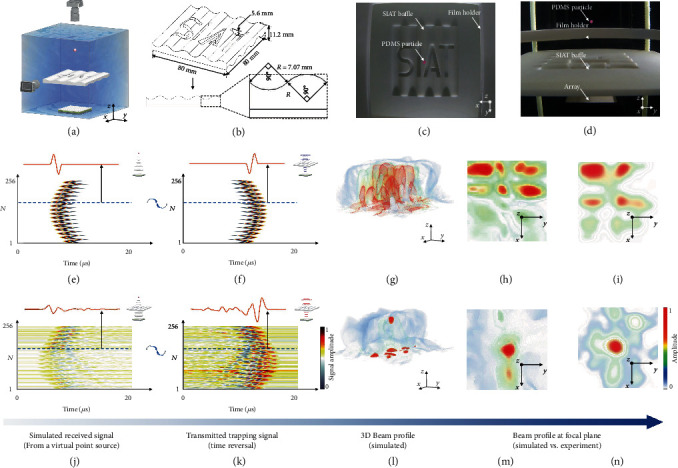
Time-reversal-based acoustic tweezers through SIAT baffle: (a) schematic of trapping the PDMS particle through the SIAT baffle using the time-reversal-based method; (b) the geometric dimensions of the SIAT baffle; (c, d) optical images of the trapped particle (dyed red) through the baffle in the top and side view, respectively; (e, j) simulated received signals of all elements from a virtual point source in the free field and through the baffle, respectively; (f, k) (f, k) transmitted trapping signals obtained from signals (e) and (j) using the time-reversal-based method, respectively; (g, l) simulated 3D beam profiles are generated after exciting the array with the signals (f) and (k), respectively, to emit acoustic waves through the SIAT baffle; (h, m) simulated beam profiles at the focal plane (60 mm from the array surface) in (g) and (l), respectively; (i, n) experimental results corresponding to (h) and (m), respectively. The signals of the same channel (No. 168) were extracted for comparison in (e–j) and (k). Particle trapping through the baffle is shown in Movie S3. Scale bars in (h), (i), (m), and (n) are 5 mm, and the rest are 10 mm.

Heterogeneity along acoustic travel paths defocuses ultrasound beams, making it challenging to implement acoustic tweezers in complex media. To mitigate this challenge, we applied the concept of time reversal in beamforming and restored beam focusing in heterogeneous media (see Materials and Methods). To validate this method, we made a complex-shaped baffle (Figure 4(b)), through which the focused beams formed assuming that the homogeneity of media (Figure 4(f)) were shown to defocus because of the phase abbreviation and distortion induced by the baffle (Figures 4(g)–4(i)). Shown in Figure 4(j) was the simulated signals originated from a target focal point located behind the baffle, received by our 256-element matrix array located on the other side of the baffle. Compared with Figure 4(e), where the baffle was absent, the waveforms and wavefronts in Figure 4(j) were distorted due to the heterogeneity. When we transmitted the signals back to the media in a time-reversed order (Figure 4(k)), the beams were refocused at the target focal spot (Figures 4(l)–4(n)), showing the effectiveness of the time-reversal-based beamforming in heterogeneous media. As an experimental validation, we showed in Figures 4(c) and 4(d) and Movie S3 that a PDMS particle placed behind the baffle was successfully trapped using the time-reversal-based method. The particle cannot be trapped with beams formed assuming homogeneity of media in the water.

**Figure 5:**
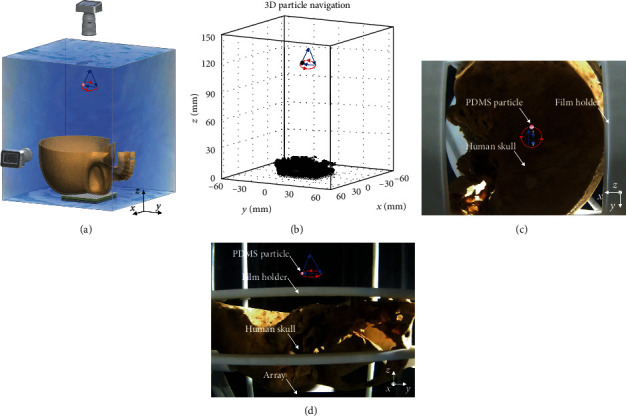
Time reversal acoustic tweezers through *ex vivo* human skull: (a) schematic of time reversal based acoustic tweezers through *ex vivo* human skull, which manipulates the PDMS particle in a 3D path (particle is transferred along an isosceles triangular path (blue curve) with a base length and height of 10 mm in the *y*-*z* plane and then along a circular path (red curve) of diameter 10 mm in the *x*-*y* plane); (b) 3D ultrasonic image of manipulated particle and schematic of motion trajectory in 3D space; (c, d) optical images of the manipulated particle (dyed red) through the skull and schematic of motion trajectory in top view (c) and side view (d). Time-reversal-based acoustic tweezers through the *ex vivo* human skull are shown in Movie S3. All scale bars are 10 mm.

Further, we implemented the time-reversal-based acoustic tweezing through an *ex vivo* human skull (Figures 5(c) and 5(d)). Shown in Figure 5(a)was the computed tomography (CT) reconstruction of the skull, which was input into our simulation that outputted the transmit waveforms we used in the experiment. In the experiment, we predefined a 3D manipulation path, which the particle was transferred along an isosceles triangular path in the *y*-*z* plane and then along a circular path in the *x*-*y* plane (Figure 5(a)). We also interleaved imaging pulses between the trapping beams and produced volumetric ultrasound images (Figure 5(b)) as a feedback of the particle manipulation process. Cross-validated by the optical imaging shown in Figures 5(c) and 5(d) and Movie S3, we manipulated a PDMS particle to move along the preset path through the skull; the distance between each movement step was 1 mm, and the time interval between steps was 0.5 second. Using the same approach, we also replicated the experiment through an *ex vivo* macaque skull (see Figure S10(a–d) and Movie S3).

## 3. Discussion

In previous studies, 3D airborne array-based acoustic tweezers were developed for the manipulation of multiple particles. Three-dimensional underwater acoustic tweezers have a higher practical application value because the acoustic impedance of water is similar to that of human tissue. Further, the unique advantage of ultrasound in water is its imaging capability without the requirement of an optically transparent medium. Ultrasonic waves attenuate rapidly in air, which makes imaging by reflecting or transmitting signals difficult. The unique imaging ability of acoustic tweezers can be used for particle navigation during trapping. Usually, selective acoustic manipulation requires the artificial placement of particles near traps, which is exceedingly difficult, especially for cells, bacteria, or other small particles. With the help of ultrasonic imaging, freely falling PDMS particles can be trapped at any initial location and then transferred to the specified position. This offers a feasible and convenient method for selective acoustic manipulation, which is more suitable for practical applications. Moreover, by combining ultrasound imaging, the volumetric ultrasonic images of particle manipulation along the 3D path through the *ex vivo* human skull can be obtained (Figure 5(b)), which indicates that the 3D ultrasonic images can record the entire manipulation process in the heterogeneous medium. Despite resolution limitations constrained by the low driving frequency and structure of the transducer, we can also observe the motion state and position of the particle through the skull. These results demonstrated the possibility of using acoustic tweezers in *in vivo* applications.

In this study, the system's response time from one frame of ultrasonic images to the next frame mainly includes the time for the transmission and reception of imaging pulses, transmission of manipulation pulses, data transfer, and image reconstruction and processing. This will greatly influence the tracking of particle manipulation. Here, we used the Verasonics Vantage 256 system (Verasonics Inc., WA, USA) as the electronic system, which has a sufficiently high system clock frequency (250 MHz) and pulse repetition rate (up to 10 kHz). First, during the transmission and reception of imaging pulses, as shown in Figure S1, four images can be obtained by transmitting 16 imaging pulses (1 cycle each pulse) with an interval of 327 *μ*s (the total transmitting time was approximately 5.2 ms). This time interval is determined by the maximum imaging depth (132 mm) and the acoustic velocity, and it is independent of the working frequency of the array. Second, during the transmission of manipulation pulses, in one manipulation sequence, ninety trapping pulses (5 cycles each pulse) were transmitted with an interval of 360 *μ*s (the total time was approximately 32.8 ms). This time interval can be adjusted for different moving steps and speed requirements under the stable manipulation condition. Third, the time required for data transfer, as well as image reconstruction and processing, largely depends on the working frequency of the array, system hardware condition, and imaging method and scope. A higher working frequency can generate smaller focal spots, making it possible to manipulate smaller particles; however, this requires a high system sampling rate, which may increase the size of the stored data, thus reducing the speed of data transfer and image reconstruction and processing. The working frequency of the array was 1.04 MHz, and the sampling rate was 4.16 MHz (quadruple sampling). When the working frequency is increased, for example, by five times, the sampling frequency should also be set to increase by five times to ensure the sampling accuracy, which increases the length of the radio frequency data to five times the original length. However, the attenuation of ultrasound will become larger with the increase in the working frequency. A smaller maximum imaging depth is usually set for higher frequencies, which reduces the number of pixels in the imaging space. Therefore, the total time of data transfer and image reconstruction and processing for high working frequencies depends on both the sampling frequency and imaging depth. The hardware condition such as the RAM memory and CPU or GPU speed of the electronic system can also be upgraded to improve the total time. The expansion of RAM space is helpful for decreasing the time required to read and store data in the real-time imaging process. The use of GPU or CPU parallel computing can improve the speed of imaging reconstruction. Further, the imaging algorithm can be optimized by reducing the numbers of plane wave compounding, and the scope of imaging acquisition can also offer some improvements, thus reducing the transmitting and receiving time of imaging, as well as the time for data transmission and reconstruction.

As shown in the results, when manipulating ten PDMS particles in a plane, the distance and time steps between the movements of each particle were approximately 1 mm and 1 s, respectively. The minimum distance between particles was approximately 5 mm. When the distance between two particles was smaller, the focal spots merged and hindered independent manipulation. The Rayleigh limit [41] for this configuration was approximately 2.5 mm (1.22*λ* *F*/*A*, where *λ* =1.5 mm, F = 60 mm, and *A* = 44.8 mm denote the wavelength, focal distance, and aperture of the array, respectively); however, the ideal distance between traps can only be achieved with a smaller array pitch (≤0.5*λ*). Moreover, we studied the relationship between the acoustic radiation force exerted on PDMS particles and the number of focal spots and elements, respectively. The simulations indicated that the maximum acoustic radiation force was inversely proportional to the number of focal points (Figure S4)), and it can be increased by reducing the pitch of the array (Figure S5). However, no further improvements were observed when the pitch of the array was smaller than *λ*/2. Both the axial and transverse radiation forces followed the same trend; however, the axial forces were smaller because of the smaller axial acoustic field gradient.

We designed a low-frequency (1.04 MHz) array with a large pitch (2*λ*) for the experiments to achieve an array with a large aperture (30*λ*) that can provide a large manipulation space and trapping force, thereby enabling *in vivo* applications. The size of the controllable particles is related to the frequency of the array; based on the low-frequency array and size of the traps (the diameters of the focal spot and vortex centre were approximately 3 and 1 mm, respectively), we selected the PDMS and PS particles with a millimetre scale for manipulation. The results also demonstrated that the trapping force and Rayleigh limit can be improved by decreasing the pitch of the array; the pitch should be reduced to near or less than *λ*/2 if possible. However, when the aperture is invariant, a smaller pitch means more array elements, which imposes new challenges to the design and manufacture of arrays and the support of excitation systems. In this study, under the premise of obtaining sufficient trapping force and manipulating space, we selected a pitch of 2*λ* to overcome the trade-off between the small pitch and the existing excitation systems and the manufacturing technology of the array. The proposed method of single-array acoustic manipulation was found to be superior compared to array-based acoustic tweezers that use the standing wave methods in terms of setup flexibility and cost reduction. Further, our method prevented the appearance of secondary nodes along the *z*-axis, which resolved a pressing problem in holographic acoustic tweezers [31]. However, there were limitations to our method. Particle manipulation in the beam propagation direction was less accurate and stable because the axial restraint only depended on the balance of gravity, buoyancy, and acoustic radiation force, while the axial radiation force was exerted upwards instead of converging at the trapping position. The instability of motion was also evident in the heterogeneous medium, where the particles floated upwards and downwards in the axial direction during movement. The distances from the transducer plane to the stably trapped particles are related to the preset focal distance and the energy exerted on the particles. In this study, the preset focal depth from 4 cm to 12 cm (beam width 2.1 mm to 3.3 mm) was selected for trapping to reduce the impact of the grating lobes and to maintain the good focusing ability of the array (Figure S6). The relationships between the driving voltage, preset focal distance, and actual trapping position were explored with particles of different sizes (0.61*λ*, 0.93*λ*, and 1.41*λ*), respectively. As shown in Figure S7, when the focal distance is constant, the trapped particles move upward with an increasing voltage. Once the driving voltage is increased to a certain extent, the particles begin to vibrate and cannot be trapped stably. By adjusting the combination of the preset focal distance and the driving voltage, the particles may be trapped more accurately at a specified height. The results also indicated that the maximum and the minimum distances from the transducer plane to the particles that can be trapped were 17.1 cm and 3.7 cm, respectively. The relationships among the duty factor, pulse repetition frequency (PRF), and position of the manipulated particle had also been studied. The duty factor used in this paper was 1.3%, and the PRF was 2.7 kHz. The influence of the duty factor on manipulation is mainly reflected in the energy that is exerted on the manipulated particles. When the PRF is constant, the particles are driven to move up with the increasing duty factor owing to the increased energy (Figure S8). However, when the duty factor is constant, increasing PRF has little influence on the trapping position (Figure S9).

In this study, a self-designed 1.04 MHz, 256-element 2D matrix array was fabricated for the underwater 3D acoustic tweezers. Its ability of 3D ultrasonic imaging in water was successfully used for guiding and monitoring the process of particle acoustic manipulation in real time. The 3D images tracked a freely falling soft particle from an arbitrary initial position and guided the system to trap and manipulate the particle according to the designed route. Moreover, we dynamically manipulated multiple relatively soft and rigid microparticles in 3D using different traps (multifoci and vortex traps). Furthermore, we introduced the time-reversal-based method to correct the phase and amplitude distortion caused by a heterogeneous medium and successfully manipulated the soft particles through a SIAT baffle and *ex vivo* macaque and human skulls. These results preliminarily address the existing challenges in the *in vivo* applications of acoustic tweezers and facilitate their development for practical biomedical applications. Future studies will be performed by exploring new acoustic traps and using arrays with higher frequencies, higher number of elements, smaller pitch, or higher transmitting output power, which will enable the manipulation of considerably smaller particles or cells and improve the capabilities of acoustic tweezers.

## 4. Materials and Methods

### 4.1. System Setup

As shown in Figure 1, we realized 3D acoustic tweezers in water using a self-fabricated 1.04 MHz, 256-element (16 × 16) ultrasonic 2D matrix array with a pitch of 2.8 mm and kerf of 0.2 mm. Each element contained matching layers, piezocomposites, Cr/Au electrodes, flexible circuits, and backing layers. The 3D acoustic tweezer system was a programmable 256-channel pulser-receiver system designed by Verasonics Vantage 256 system. The system was used for exciting arrays using trapping and imaging pulse signals to obtain specified acoustic fields and for receiving echo signals to reconstruct images. The phase, amplitude, and waveform of a signal exerted on each element can be adjusted dynamically and independently. The navigation system referred to the high-performance workstation T5810 (Dell Inc., TX, USA) capable of running simulation programs, which dictated acoustic tweezer beamforming by calculating the specified pulse signals and guiding the 3D acoustic tweezer system to excite the array according to the signals.

#### 4.1.1. Self-Navigated 3D Acoustic Tweezers

The matrix array was first driven by imaging pulses. The echo signals from the particle were reconstructed for the 3D ultrasound images to indicate the current location of the particle (Figure 1, *P*_0_ position). The navigation system dictated the trapping beams to seize particles by importing the position information in real time (Figure 1, black loops in the middle). The imaging sequences were interleaved between the manipulation sequences (see Figure S1 and Note S3 for details regarding sequence arrangement). The on-time duration of the half cycle and the number of cycles for each pulse signal in these two sequences were different; a higher on-time duration (0.9) and greater number of cycles (five cycles) provide higher energy in the manipulation sequence, while a lower on-time duration (0.8) and smaller number of cycles (one cycle) provide higher resolution in the imaging sequence. The transmitting signals of each element in the manipulation sequence were different, and the generated sound waves converged to form the target multifoci acoustic field. When trapped, the particle can be transferred along a user-defined path by changing the position of the focal spots. The path was divided into a finite number of steps by the navigation system, and the distance of each step of the particle movement was approximately 0.5 mm. The CPWC method was used for the imaging sequence, which improved the image quality of the single plane wave imaging mode [40]. Four plane waves with different inclination angles were transmitted, and each frame of the 3D image was obtained by coherently compounding the images obtained with the waves. The entire 3D imaging space of the self-navigated 3D acoustic tweezers is shown in Figure S2.

#### 4.1.2. Three-Dimensional Acoustic Tweezers for Multiple Particles

In a homogeneous medium, the navigation system calculated the appropriate phase delays using the acoustic travel time, which is equal to the distance between each element and focal spot divided by the wave velocity, and it then passed them to the 3D acoustic tweezer system. When the particle was trapped, the navigation system can divide the path into a finite number of steps by inputting the motion trajectory information (Figure 1, from *P*_0_ through *P*_1_ to *P*_2_, blue dashed line); each step can update the trapping beams to manipulate the particle along the path (Figure 1, blue loops).

Particles with different acoustic contrast factors exist in water [42]. Here, we used the PDMS and PS particles to demonstrate the capability to trap particles with negative and positive contrast factors, respectively. To manipulate the particles, we implemented multifoci (Figure S3(a)) and vortex (Figure S3(d)>) acoustic traps, respectively. The directions of the acoustic radiation force exerted on the PDMS (Figure S3(b–c)) and PS (Figure Figure S3(e–f)) particles were opposite (Note S1). The radiation force exerted on the PDMS particles at multiple focal spots and that exerted on the PS particles at the centre of the vortex converges in the lateral directions and directs upward in the axial direction, thereby enabling the trapping and manipulation of the particles. The transmission signals of each element were determined using the pseudoinverse algorithm (PINV) [43, 44] to generate the multifoci field by initializing the position of the focal spots (Note S2). The vortex was realized by adjusting the transmission phase of each element to generate a screw dislocation in the wavefront [45, 46] (Note S2). When particles were trapped at the focal spots or the vortex centre, the transmitting signals were dynamically changed to move the spots or vortex and hence, the particles. In this study, we manipulated the particles initially resting on a film, thereby enabling the acoustic waves to propagate while suffering little reflection and maintaining travelling-wave character.

We manipulated two PDMS particles in a 3D space simultaneously, whose motion trajectory was preset as a spatial ellipse (the angle between the plane of motion and *z*-axis was 45° and the diameter of the circular path in the top view was 12 mm). Ten particles were manipulated to transform a circle (diameter = 20 mm) to a pentagon (outer circle diameter = 20 mm) and rotate the circle in the clockwise and counterclockwise directions. One PS particle (diameter = 500 *μ*m) was trapped to move in a circular path (diameter = 10 mm). A large number of PS particles (diameter = 400–800 *μ*m) were manipulated to display the patterns of the vortex (topological charge = 1), pentagon (40 closely arranged focal spots, outer circle diameter = 24 mm), and snowflake (48 closely arranged focal spots, outer circle diameter = 24 mm) acoustic fields.

#### 4.1.3. Time-Reversal-Based Acoustic Tweezers in Heterogeneous Media

To achieve acoustic manipulation in heterogeneous media, the trapping beams should be carefully designed by considering the possible phase abbreviations or distortions caused by the media. The navigation system can simulate the wave propagation process by importing the medium information (Figure 1, red dashed line) and perform beamforming using signals generated by the time-reversal-based method. This enables the trapping beams to focus at the target position (Figure 1, red loops).

The time-reversal-based method was used for the phase and amplitude correction [47]. The steps in the method are as follows: (1) use the array and heterogeneous medium information (e.g., density, acoustic velocity, and geometry) for acoustic wave propagation modelling based on the navigation system and simulate ultrasound wave signals which propagate through the medium by setting up a virtual sound source at the target trapping position; (2) reverse the simulated signals received by each element along the timeline, and divide them by the amplitude correction coefficient which is equal to the maximum signal amplitude of each element divided by the maximum signal amplitude of all elements; and (3) obtain the transmitting signals by extracting the phase, amplitude, and waveform information of the reversed and modified simulated signals. When the array was driven by the transmitting signals, the acoustic waves propagated through the heterogeneous medium and refocused at the position of the original virtual sound source. Then, the PDMS particles were trapped at the focal spot in the heterogeneous medium.

In this study, we successively added a SIAT baffle, an *ex vivo* macaque (Figure S10(a)), and an *ex vivo* human skull between the array and the PDMS particle to demonstrate the heterogeneous medium. The SIAT baffle was 3D-printed with photosensitive resin (Figure 4(b)). The SIAT baffle had the following dimensions: 80 mm × 80 mm × 11.2 mm. Wave stripes were positioned perpendicular to each other on the upper and lower surfaces (the radius of curvature of each wave stripe was 7.07 mm, and the central angle was 90°), and SIAT grooves were engraved on the wave stripes on the top surface (the bottom of the groove was 5.6 mm away from the top surface) to add more irregularities to the baffle. The sound velocity and density of the baffle were measured to be 2368 m/s and 1123 kg/m^3^, respectively. The absorption parameter was measured to be 7.3 dB/cm at 1.04 MHz. The designed SIAT baffle can be directly modelled in the simulation by importing information related to its geometry and the acoustic parameters. The acoustic pressure maps obtained through the SIAT baffle with and without applying the time-reversal-based method were simulated and measured to explore the effectiveness of the method.

The macaque and human skulls were obtained from the corpses of a macaque and human, respectively, following Chinese ethical laws. The macaque skull was cut transversely, whereas the human skull was cut longitudinally and opened using a saw. The skulls were prepared by adopting standard anatomical procedures; they were cleaned and disinfected subsequently. The *ex vivo* macaque skull had dimensions of 65 mm × 68 mm × 38 mm and an irregular quarter spherical shell shape with an average thickness of approximately 1.5 mm. We used the right half of the *ex vivo* human skull with dimensions of 19 cm × 14 cm × 8 cm and average thickness of approximately 12 mm; this was approximately eight times thicker than the *ex vivo* macaque skull. Before the experiment, the skulls were immersed in degassed water for over 48 h. The experiments were performed in the degassed water at room temperature.

k-Wave, a MATLAB toolbox for the time-domain simulation of acoustic wave fields, was used to perform all simulations in the heterogeneous medium [48]. The core advantage of k-Wave is its advanced numerical model that can account for the arbitrary distribution of heterogeneous medium parameters along with linear and nonlinear wave propagation.

In the case of acoustic manipulation through the skulls, because the geometry of the skulls was not designed by us, we used CT to scan the entire setup of the experiment (including the array, skulls, and bracket); next, we imported the obtained relative position information of the skulls into the simulation. The sound velocity and density information at each simulated grid point of the skulls was obtained by mapping the CT values of the corresponding pixels in the CT images [49]. When excluding the unnecessary parts, we set the minimum CT value corresponding to the sound velocity and density (3100 m/s and 2200 kg/m^3^, respectively) of the skull and the maximum CT value corresponding to the sound velocity and density (1540 m/s and 1000 kg/m^3^, respectively) of water. At the simulation points where the CT values varied between the maximum and minimum values, the sound velocity and density information can be obtained by linearly corresponding to the sound velocity (1540–3100 m/s) and density intervals (1000–2200 kg/m^3^), respectively.

### 4.2. Design and Fabrication of 2D Matrix Transducer

The 256-element (16 × 16) 2D matrix array was fabricated using the conventional transducer technology. A piece of PZT-5H bulk ceramic was used to prepare the piezoelectric 1-3 composite based on a dice-and-fill technique. In this study, a 170 *μ*m thick dicing blade was selected to achieve a kerf of 200 *μ*m and a pitch of 700 *μ*m. Then, the prepared PZT-5H 1-3 composite was lapped down to the designed thickness based on the PiezoCAD simulation result. Both polished surfaces were sputtered with a chrome/gold (Cr/Au) electrode using a sputtering system with the designed thicknesses of 200 nm/500 nm. The front side of the PZT-5H composite was cast with a matching material made of aluminium oxide powder (23 *μ*m) and Epo-Tek 301 epoxy (Epoxy Technologies, MA, USA). After curing at room temperature for 24 h, the matching layer was polished to an optimized thickness of 750 *μ*m. The prepared acoustic stack with the matching layer was diced into the designed dimensions of 44.8 mm × 44.8 mm, which consisted of 256 array elements. Then, the back side of the PZT-5H 1-3 composite was diced with an electrode division kerf of 200 *μ*m, a depth of 200 *μ*m, and an element width of 2.6 mm × 2.6 mm for the array assembly process. Four pieces of customized flexible circuits fabricated using polyimide with 64 electrode traces (trace width = 254 *μ*m and thickness = 118 *μ*m) were soldered to the array elements. Then, the array elements attached to the flexible circuits were fixed inside an acrylic housing, and the gap between the acoustic stack and housing was filled with an insulating epoxy. A total of 256 coaxial cables were sealed with the flexible circuit for the further characterization and application of acoustic tweezers. Finally, a backing material made of Epo-Tek 301 epoxy with 3–5 *μ*m aluminium nitride powder was filled into the housing to reduce the ring-down effect.

### 4.3. Fabrication of Particles

The employed particles were 0.9–3 mm diameter PDMS spheres and 0.4–1 mm diameter PS spheres (Huge Biotechnology Co., Ltd., Shanghai, China). The PDMS elastomer kit (Sylgard 184) was purchased from Dow Corning, and Span 80 and hexane were obtained from Sinopharm Chemical Reagent Co., Ltd. (Shanghai, China). The uniform PDMS microspheres were prepared using a modified mechanical emulsification method [50–52]. The prepolymer base elastomer and the curing agent were mixed with a weight ratio of 10 : 1 under intense stirring. Then, the obtained mixtures were diluted by hexane with a volume ratio of 1 : 9, followed by mechanical stirring and vacuum pumping for 15 min. Finally, the mixture was transferred to a syringe (10 *μ*l). Subsequently, the resulting solution was slowly injected into an aqueous solution (10 ml) that contained the surfactant, Span 80 (5% *w*/*w*), without any agitation. Finally, the obtained PDMS microspheres were cured at room temperature for 24 h.

## Supplementary Materials

Supplementary Materials Note S1: calculating the acoustic radiation force for soft and rigid particles. Note S2: generation of complex acoustic field. Note S3: self-navigated 3D acoustic tweezers. Figure S1: schematic of manipulation sequence and imaging sequence in self-navigated programmable 3D acoustic tweezers. Figure S2: schematic of imaging space in self-navigated programmable 3D acoustic tweezers. Figure S3: acoustic radiation force calculation for different particles. Figure S4: relationship between simulated maximum acoustic radiation force and number of focal spots. Figure S5: relationship between simulated maximum acoustic radiation force and number of elements under the same aperture. Figure S6: the measured beam profiles, relationship between beam width and maximum acoustic pressure, and simulated acoustic fields at different focal depths. Figure S7: relations of particle trapping position of different sizes with preset focal distance and driving voltages. Figure S8: relationship between duty factor and position of manipulated particles. Figure S9: relationship between PRF and position of manipulated particles. Figure S10: time reversal acoustic tweezers through *ex vivo* macaque skull. Table S1: simulation calculation parameters. Movie S1: self-navigated programmable 3D acoustic tweezers. Movie S2: 3D acoustic tweezers for different particles. Movie S3: time reversal acoustic tweezers in complex medium.
