# Supplementary material for: Self-Navigated 3D Acoustic Tweezers in Complex Media Based on Time Reversal
Source: Research (Wash D C). 2021 Jan 4;2021:9781394. doi: 10.34133/2021/9781394 (PMC7877394; doi:10.34133/2021/9781394)
Supplement: Supplementary Materials — Note S1: calculating the acoustic radiation force for soft and rigid particles. Note S2: generation of complex acoustic field. Note S3: self-navigated 3D acoustic tweezers. Figure S1: schematic of manipulation sequence and imaging sequence in self-navigated programmable 3D acoustic tweezers. Figure S2: schematic of imaging space in self-navigated programmable 3D acoustic tweezers. Figure S3: acoustic radiation force calculation for different particles. Figure S4: relationship between simulated maximum acoustic radiation force and number of focal spots. Figure S5: relationship between simulated maximum acoustic radiation force and number of elements under the same aperture. Figure S6: the measured beam profiles, relationship between beam width and maximum acoustic pressure, and simulated acoustic fields at different focal depths. Figure S7: relations of particle trapping position of different sizes with preset focal distance and driving voltages. Figure S8: relationship between duty factor and position of manipulated particles. Figure S9: relationship between PRF and position of manipulated particles. Figure S10: time reversal acoustic tweezers through ex vivo macaque skull. Table S1: simulation calculation parameters. Movie S1: self-navigated programmable 3D acoustic tweezers. Movie S2: 3D acoustic tweezers for different particles. Movie S3: time reversal acoustic tweezers in complex medium. [file 9781394.f1.zip › Supplementary Materials(Proof Version).pdf]

Research
A SCIENCE PARTNER JOURNAL
SPJ
SCIENCE PARTNER JOURNALS

Manuscript Template

Supplementary Materials of

"Self-navigated 3D Acoustic Tweezers in Complex Media based on Time Reversal"

Authors

Ye Yang[1,2,†], Teng Ma[1,2,†*], Sinan Li[3,†], Qi Zhang[1], Jiqing Huang[1], Yifei Liu[1,2], Jianwei Zhuang[1], Yongchuan Li[1], Xuemin Du[1,2], Lili Niu[1,2], Yang Xiao[1,2], Congzhi Wang[1,2], Feiyan Cai[1,2*], and Hairong Zheng[1,2*]

Affiliations

[1] Paul C. Lauterbur Research Center for Biomedical Imaging, Institute of Biomedical and Health Engineering, Shenzhen Institutes of Advanced Technology, Chinese Academy of Sciences, Shenzhen 518055, China

[2] Shenzhen College of Advanced Technology, University of the Chinese Academy of Sciences, Beijing 100049, China

[3] VERASONICS, INC., WA 98034, USA

[†] These authors contributed equally to this work.

[*]Correspondence should be addressed to Teng Ma; teng.ma@siat.ac.cn. Feiyan Cai; fy.cai@siat.ac.cn and Hairong Zheng; hr.zheng@siat.ac.cn.

Supplementary Notes

Note S1: Calculating the acoustic radiation force for soft and rigid particles

The acoustic radiation force of a given particle can be calculated under a known sound field distribution. Neglecting thermal and viscosity effects, the acoustic radiation force can be expressed as the stress passing through the fixed surface enclosing the scatterer within a given period of time [1].

$$\mathbf{F} = -\oint_{\Sigma} \frac{d\mathbf{F}}{d\Sigma} d\Sigma , \tag{1}$$

$$\frac{d\mathbf{F}}{d\Sigma} = \left[\frac{1}{2\rho_0 {c_0}^2} \left\langle p^2 \right\rangle - \frac{\rho_0}{2} \left\langle |\mathbf{v}|^2 \right\rangle \right] \mathbf{n} + \rho_0 \left\langle (\mathbf{n} \cdot \mathbf{v}) \mathbf{v} \right\rangle , \tag{2}$$

where $\sum$ is the surface of the scatterer; ρ_0 and c_0 represent the fluid density and sound velocity, respectively; p and $\mathbf{v}$ represent the acoustic pressure and velocity, respectively; $\mathbf{n}$ is the outward unit normal vector for the surface element $d\sum$; and $\langle \cdot \rangle$ denotes the time average over a single sound wave period. The acoustic radiation force calculated by Equation (2) can be applied to particles of any size because it considers the scattering effect of particles on the sound field.

When the diameter of the spherical particle is smaller than or approximately equal to the wavelength in the medium, the influence of the particle on the sound field can be neglected. The energy density of the sound field is continuous in space; therefore, the acoustic radiation force on the particle can be considered to be approximately equal to the negative gradient of time-averaged energy density of the sound field [2, 3].

$$\mathbf{F} = -\nabla \left\langle E \right\rangle , \tag{3}$$

where the spatial energy density can be expressed as

$$E = 2\pi \rho_0 R^3 (\frac{\left\langle p^2 \right\rangle}{3\rho_0^2 c_0^2} a_1 - \frac{\left\langle v^2 \right\rangle}{2} a_2) , \tag{4}$$

$$a_1 = 1 - \frac{c_0^2 \rho_0}{c_s^2 \rho_s}, \; a_2 = 2\frac{\rho_s - \rho_0}{2\rho_s + \rho_0}, \tag{5}$$

where ρ_s and c_s denote the particle density and sound velocity, respectively, and R is the radius of the particle. When complex multi-foci or vortex acoustic fields are given or calculated, the velocity field can be computed as

$$\mathbf{v} = \nabla \mathbf{p} / i\rho_0\omega, \tag{6}$$

where $i = (-1)^{1/2}$ and ω is the angular frequency. Following this, the energy density and acoustic radiation force can be calculated for different particles using Equations (3)-(6). Here, the respective densities and sound velocities of polydimethylsiloxane (PDMS), polystyrene (PS), and fluid, which are used for calculating the acoustic radiation force, are listed in Table S1. The relocation performance of the particles is determined by their different densities (ρ_s) and compressibilities (β_s, determined by the density and both primary and secondary sound velocities) relative to the surrounding fluid. The PDMS particles (which can be considered as soft particles) have higher compressibility and lower density; they are driven by the acoustic radiation force from the weak acoustic pressure position towards the strong acoustic pressure position. Further, the PS particles (which can be considered as rigid particles) have lower compressibility and higher density; they are driven in the direction opposite to that of the PDMS particles [4-6].

Note S2: Generation of complex acoustic field

Based on the physical properties (density and compressibility) of different particles, the PDMS and PS particles were manipulated by changing the acoustic field. Complex multi-foci acoustic fields were used for trapping and translating the PDMS particles, while vortex acoustic fields were applied for trapping and translating the PS particles.

In this study, a 1.04 MHz 256-element 2D matrix array with a pitch of 2.8 mm and kerf of 0.2 mm was designed and fabricated for 3D acoustic tweezers. The array transducer was driven by the Verasonics Vantage 256 system (Verasonics Inc., WA, USA). A dynamic burst signal with different amplitude and phase was used to excite individual elements to form a complex acoustic field of multiple focal spots and vortexes to precisely trap and manipulate the PDMS and PS particles within a 3D space.

The complex acoustic fields were accurately simulated using Fast Object-Oriented C++ Ultrasound Simulator (FOCUS) [7, 8], which can be used as a toolbox of the MATLAB platform (Mathworks, Natick, MA, USA). The parameters used in the simulation, such as those of the array, density, and acoustic velocity of the medium, and the division of the computing grid were set to match those of the actual experiments.

The pseudo-inverse algorithm (PINV) was used to calculate the amplitude and phase of each element in the array for generating complex acoustic fields with multiple focal spots [9, 10]. The acoustic pressure at spatial point r can be calculated by the Rayleigh–Sommerfeld integral [11].

$$p(r)=\frac{j\rho_0 c_0}{\lambda}\int_S \mathbf{u}_{\mathbf{r}'}\frac{\exp(-jkd_{rr'})}{d_{rr'}}dS' \ , \tag{7}$$

where j = $(-1)^{1/2}$; ρ_0 and c_0 represent the medium density and sound velocity, respectively; λ and k represent the wavelength and wavenumber of the ultrasound, respectively; S' is the surface of the source; $\mathbf{u}_{r'} = |u_{r'}|e^{j\varpi t}$ is the complex velocity at point r' on the surface of the source (ϖ is the angular frequency and t denotes the time); and $d_{rr'}$ is the distance between the observation point r and source point r'. For an ultrasound array with N elements, the acoustic pressure is calculated as

$$p(r)=\frac{j\rho_0 c_0}{\lambda}\sum_{n=1}^{N}\mathbf{u}_{\mathrm{n}}\int_{S_n'}\frac{\exp(-jkd_{rr_n'})}{d_{rr_n'}}dS_n' \ , \tag{8}$$

where r_n is the centre point on the nth element of the array, and S_n' and $\mathbf{u}_\mathrm{n}$ represent the surface and complex velocity of the nth element, respectively. If there are M focal spots, the complex pressure at focal spot r_m becomes

$$p(r_\mathrm{m}) = \frac{j\rho_0 c_0}{\lambda} \sum_{n=1}^{N} \mathbf{u}_\mathrm{n} \int_{S_n'} \frac{\exp(-jkd_{r_m r_n'})}{d_{r_m r_n'}} dS_n' \quad (9)$$

,

$m = 1, 2, ..., M.$

This equation can also be written in the matrix form as

$$\mathbf{Hu} = \mathbf{p}\,, \quad (10)$$

where $\mathbf{u} = [u_1, u_2, ... , u_n]$' represents the complex excitation vector of the array elements; $\mathbf{p} = [p(r_1), p(r_2), ... , p(r_m)]$' is the complex pressure at focal spots; and $\mathbf{H}$ denotes the forward propagation operator with elements.

$$H(m,n) = \frac{j\rho_0 c_0}{\lambda} \int_{S_n'} \frac{\exp(-jkd_{r_m r_n'})}{d_{r_m r_n'}} dS_n'\,, \quad (11)$$

If the multi-foci complex pressure $\mathbf{p}$ is known or specified, the excitation vector of the elements of the array can be calculated using

$$\mathbf{u} = \mathbf{H}^{*t} (\mathbf{HH}^{*t})^{-1} \mathbf{p}\,, \quad (12)$$

where $\mathbf{H}^{*t}$ is the conjugate transpose matrix of $\mathbf{H}$.

Thus, the transmitting phase and amplitude of each element can be calculated by simulating the specified multiple focal spots, following which, the excitation system correspondingly drives the elements in the experiment with these data, thereby generating the target multiple focal spots in the space. As previously stated, the direction of the radiation force exerted on the PDMS particles will trap them at the focal spots of the multi-foci field. Therefore, the PDMS particles can be continuously manipulated by dynamically controlling the position of multiple focal spots in real time.

The acoustic field of the vortex is set by adjusting the transmitting phase of the elements of the transducer array to generate a screw dislocation in the wavefront [12, 13]. The phase of the output must be a function of the angle about the centre of the target vortex, which is expressed as

$$\theta_{OAM} = l \times \varphi + r \times \alpha \quad , \tag{13}$$

where θ_{OAM} is the transmitting phase of the elements, l represents the topological charge of the vortex field, α is the curl of the phase, and φ and r denote the azimuth angle and normalized radial coordinates about the centre of the target vortex, respectively. Additionally, the transmitting amplitudes of all elements were set to the same value. The position of the vortex field can be moved by adjusting the central position, which is related to φ and r in phase calculation. In this study, $l = 1$ was set for phase increase in the counterclockwise direction, and α was set to 10 for the appropriate curl of phase distribution. The vortex acoustic field was used to manipulate the PS particles. A phase singularity exists at the centre of the vortex where the amplitude is zero and the phase is not defined; the PS particle can be trapped at the centre of the vortex and driven to move by changing the position of the vortex.

Note S3: Self-navigated 3D acoustic tweezers

For the manipulation sequence, each transmitting pulse was set with five cycles at a driving frequency of 1.04 MHz, and a total of 90 pulses were transmitted within 360 μs of the pulse interval. The focus wave beams calculated by the result of the multi-foci simulation were used in the manipulation sequence. For comparison, each transmitted pulse in the imaging sequence was set with one cycle at the same frequency. Four pulses were transmitted at pulse intervals of 327 μs to reconstruct a frame image, and a total of four frame images were acquired (Figure S1).

The entire 3D imaging space is a flat top pyramid, which can be considered to be a pyramid shape (the apex of the pyramid lies on the negative z-axis) truncated by two planes parallel to the x-y plane. The lower plane of the flat top pyramid is the plane of the array surface, and the upper plane (with a length and width of approximately 132 mm (88 λ) each) is approximately 132 mm (88 λ) away from the lower plane (Figure S2).

Supplementary References

[1] P. J. Westervelt, "The theory of steady forces caused by sound waves". *J. Acoust. Soc. Am.*, vol. 23, no. 3, pp. 312–315, 1951.

[2] A. P. Sarvazyan, O. V. Rudenko, W. L. Nyborg, "Biomedical applications of radiation force of ultrasound: historical roots and physical basis". *Ultrasound Med. Biol.*, vol. 36, no. 9, pp. 1379–1394, 2010.

[3] L. V. King, "On the acoustic radiation pressure on spheres". *Proc. Roy. Soc. Lon. Ser. A*, vol. 147, no. 861, pp. 212–240, 1934.

[4] L. P. Gor'kov, "On the forces acting on a small particle in an acoustical field in an ideal fluid". *Sov. Phys. Dokl.*, vol. 6, no., pp. 773–775, 1962.

[5] H. Bruus, "Acoustofluidics 7: The acoustic radiation force on small particles". *Lab Chip*, vol. 12, no. 6, pp. 1014–1021, 2012.

[6] L. M. Johnson *et al.*, "Elastomeric microparticles for acoustic mediated bioseparations". *J. Nanobiotechnol.*, vol. 11, no., pp. 22, 2013.

[7] R. J. McGough, "Rapid calculations of time-harmonic nearfield pressures produced by rectangular pistons". *J. Acoust. Soc. Am.*, vol. 115, no. 5 Pt 1, pp. 1934–1941, 2004.

[8] D. Chen, R. J. McGough, "A 2D fast near-field method for calculating near-field pressures generated by apodized rectangular pistons". *J. Acoust. Soc. Am.*, vol. 124, no. 3, pp. 1526–1537, 2008.

[9] E. S. Ebbini, C. A. Cain, "Multiple-focus ultrasound phased-array pattern synthesis: optimal driving-signal distributions for hyperthermia". *IEEE Trans. Ultrason. Ferroelectr. Freq. Control*, vol. 36, no. 5, pp. 540–548, 1989.

[10] Y. Hertzberg, O. Naor, A. Volovick, S. Shoham, "Towards multifocal ultrasonic neural stimulation: pattern generation algorithms". *J. Neural Eng.*, vol. 7, no. 5, pp. 056002, 2010.

[11] A. Weyns, "Radiation-field calculations of pulsed ultrasonic transducers .1. Planar circular, square and annular transducers". *Ultrasonics*, vol. 18, no. 4, pp. 183–188, 1980.

[12] B. T. Hefner, P. L. Marston, "An acoustical helicoidal wave transducer with applications for the alignment of ultrasonic and underwater systems". *J. Acoust. Soc. Am.*, vol. 106, no. 6, pp. 3313–3316, 1999.

[13] C. Shi, M. Dubois, Y. Wang, X. Zhang, "High-speed acoustic communication by multiplexing orbital angular momentum". *Proc. Natl. Acad. Sci. USA*, vol. 114, no. 28, pp. 7250–7253, 2017.

Supplementary Tables

Table S1. Simulation calculation parameters

Parameter	Density (kg/m^3)	Sound Velocity (m/s)	a_1	a_2
PDMS	1274	1071	-0.6229	0.1545
PS	2200	2337	0.8026	0.4444
Fluid	1000	1540	-	-

Supplementary Figures

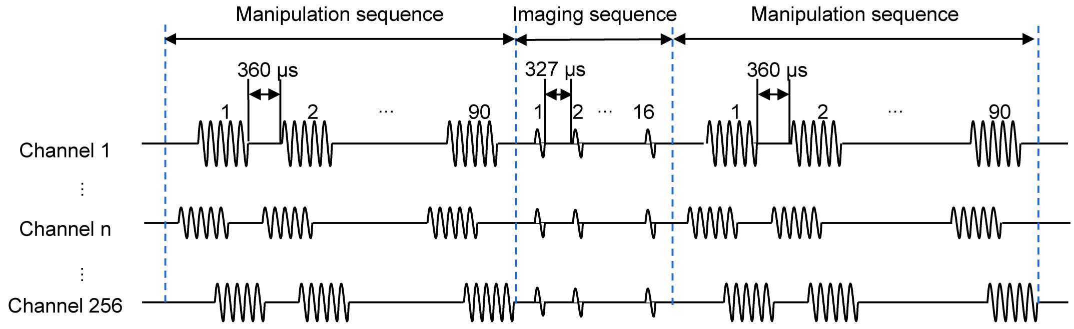

FIGURE S1: Schematic of manipulation sequence and imaging sequence in self-navigated programmable 3D acoustic tweezers.

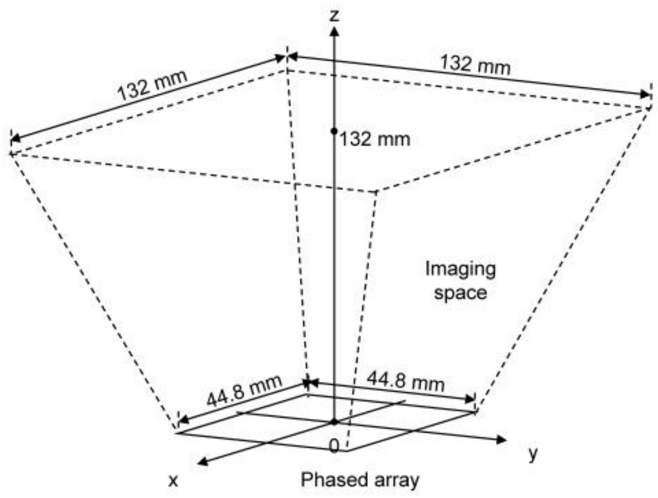

FIGURE S2: Schematic of imaging space in self-navigated programmable 3D acoustic tweezers.

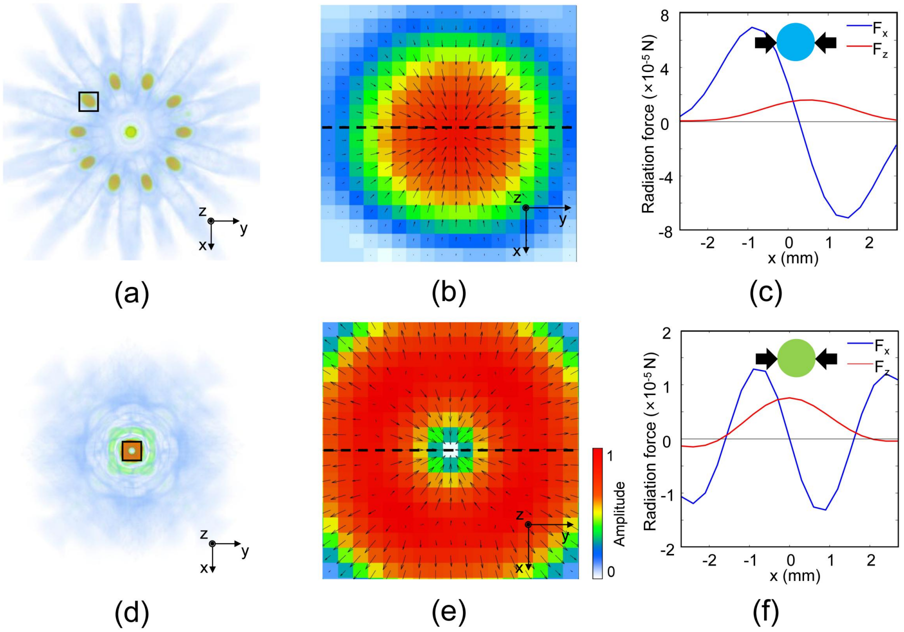

FIGURE S3: Acoustic radiation force calculation for different particles**. (**a), Simulated 3D beam profile of the multi-foci acoustic field in which ten focal spots are distributed on a circle with a radius of 10 mm. (b), Details of simulation acoustic pressure and radiation force vectors of the PDMS particle at the focal point (focal depth = 60 mm), where the black square in (a) marks the area of (b). (c), Acoustic radiation forces F_x and F_z along the dashed cut line in (b). (d), Simulated 3D beam profile of the vortex acoustic field. (e), Details of simulation acoustic pressure and radiation force vectors of the PS particle at the vortex (60 mm away from the array surface), where the black square in (d) marks the area of (e). (f), Acoustic radiation forces F_x and F_z along the dashed cut line in (e). Scale bars in (a) and (d) are 5 mm, and the rest are 0.5 mm.

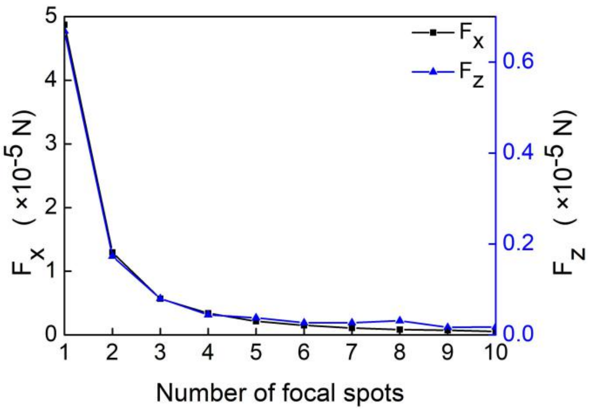

FIGURE S4: Relationship between simulated maximum acoustic radiation force and number of focal spots. Simulated maximum acoustic radiation force in the x- and z-directions depending on the number of focal spots of a 44.8 mm × 44.8 mm array (16 × 16 elements). One to ten focal spots are randomly generated in a 30 mm × 30 mm × 30 mm cube, whose centre is on the central axis of the array and distance from the surface of the array is 60 mm (16 × 16 with 2.8 mm elements and pitch). The minimum distance between any two focal points is greater than 8 mm). Radiation forces in the x- and z-directions follow similar trends, and the force in the z-direction is generally smaller, primarily owing to the smaller axial gradient.

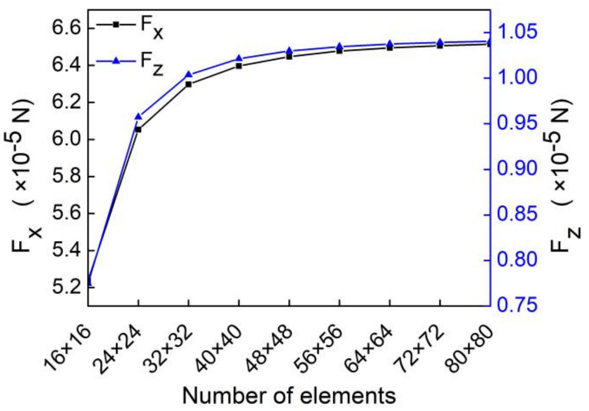

FIGURE S5: Relationship between simulated maximum acoustic radiation force and number of elements under the same aperture. Simulated maximum acoustic radiation force in the x- and z-directions depending on the number of elements in a 44.8 mm × 44.8 mm array (16 × 16, 24 × 24, 32 × 32, 40 × 40, 48 × 48, 56 × 56, 64 × 64, 72 × 72, and 80 × 80 elements). The focus is on the central axis of the array and its distance from the surface of the array is 60 mm. It should be noted that after obtaining an array with 64 × 64 elements, there is no further improvement because the element pitch is already smaller than $\lambda/2$. Radiation forces in the x- and z-directions followed similar trends, and the force in the z-direction is generally smaller, primarily owing to the smaller axial gradient.

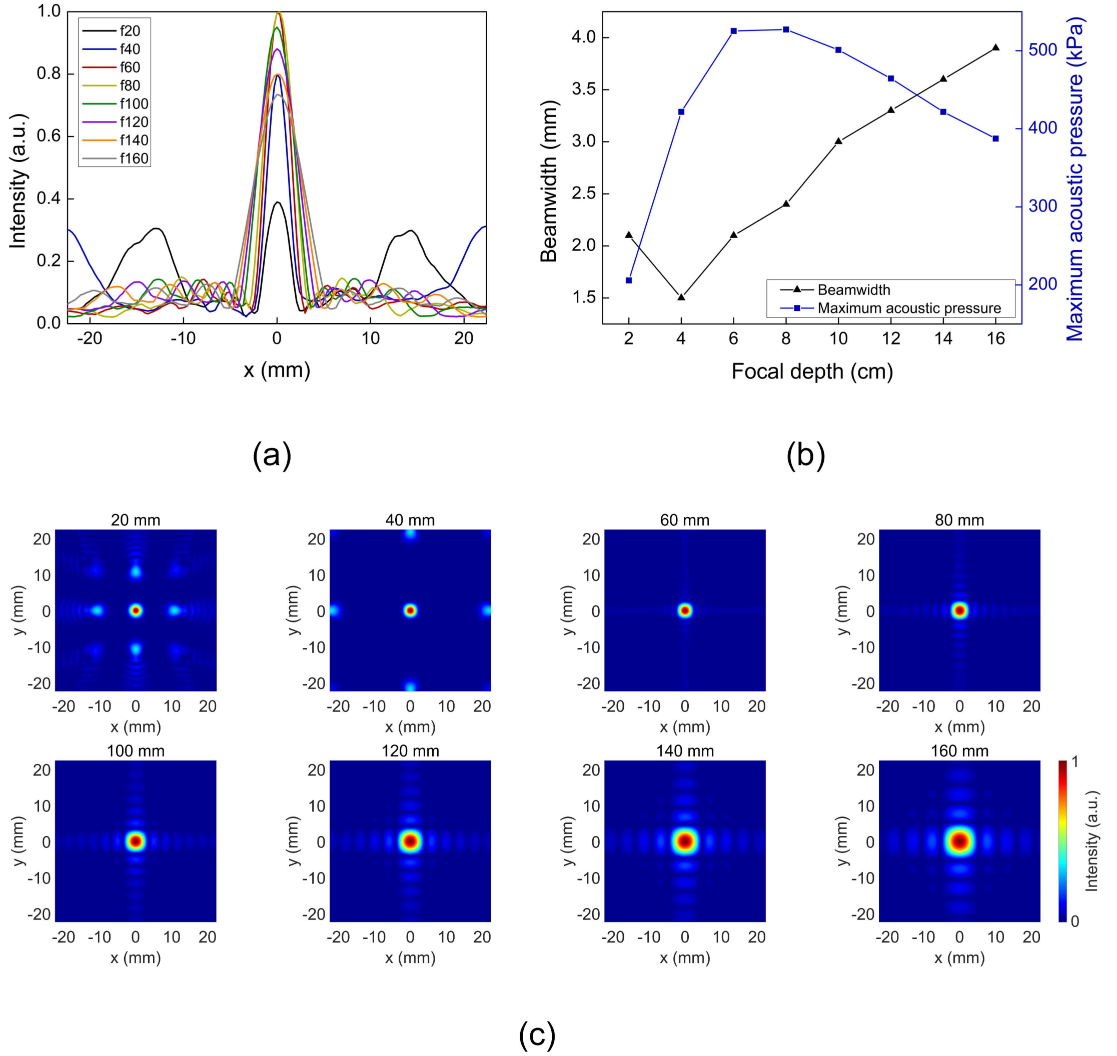

FIGURE S6. The (a) measured beam profiles, (b) relationship between beam width and maximum acoustic pressure, and (c) simulated acoustic fields at different focal depth.

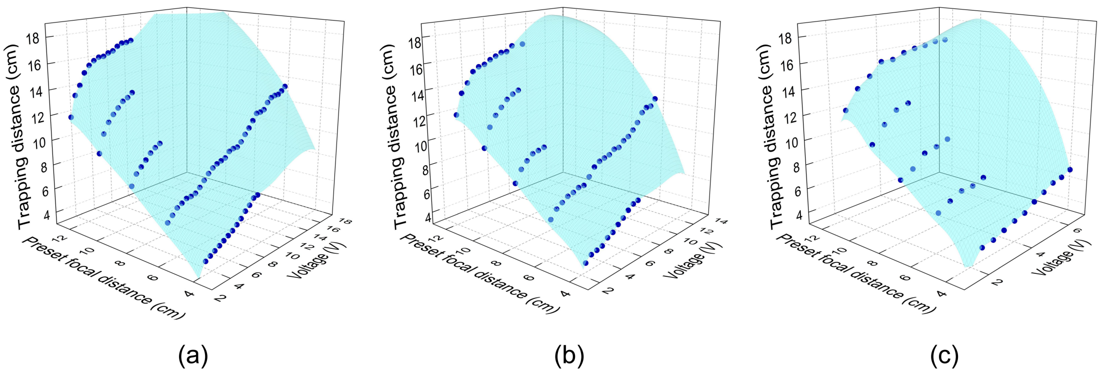

FIGURE S7. Relations of particle trapping position of different sizes ((a) 1.41 λ, (b) 0.93 λ, and (c) 0.61 λ) with preset focal distance and driving voltages. Blue dots represent the actual trapping positions which are measured experimentally under the conditions of corresponding voltages and preset focal distance. The surfaces are obtained by fitting these measured points.

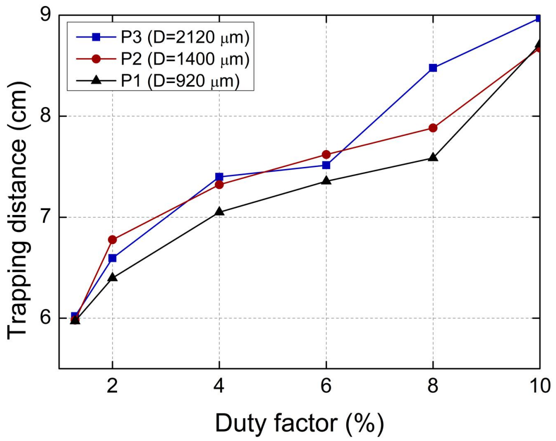

FIGURE S8. Relationship between duty factor and position of manipulated particles (PRF = 2.7 kHz). At 1.3% duty factor, the minimum voltages that can trap three particles of different diameters (0.61 λ, 0.93 λ, 1.41 λ) at 60 mm focal depth are explored and are found to be 2 V, 2.6 V and 3.3 V, respectively. The three differently sized particles are trapped under the respective minimum voltages and the distances that offset the preset focus present an increasing trend with increasing duty factor, with the PRF constant.

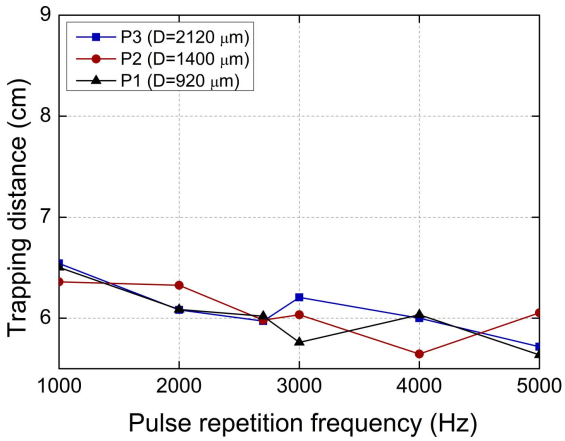

FIGURE S9. Relationship between PRF and position of manipulated particles (duty factor = 1.3 %). At 1.3% duty factor, the minimum voltages that can trap three particles of different diameters (0.61 λ, 0.93 λ, 1.41 λ) at 60 mm focal depth are explored and are found to be 2 V, 2.6 V, and 3.3 V, respectively. The three differently sized particles are trapped under the respective minimum voltages and the distances that offset the preset focus present a trend of little change with increasing PRF, with the duty factor constant.

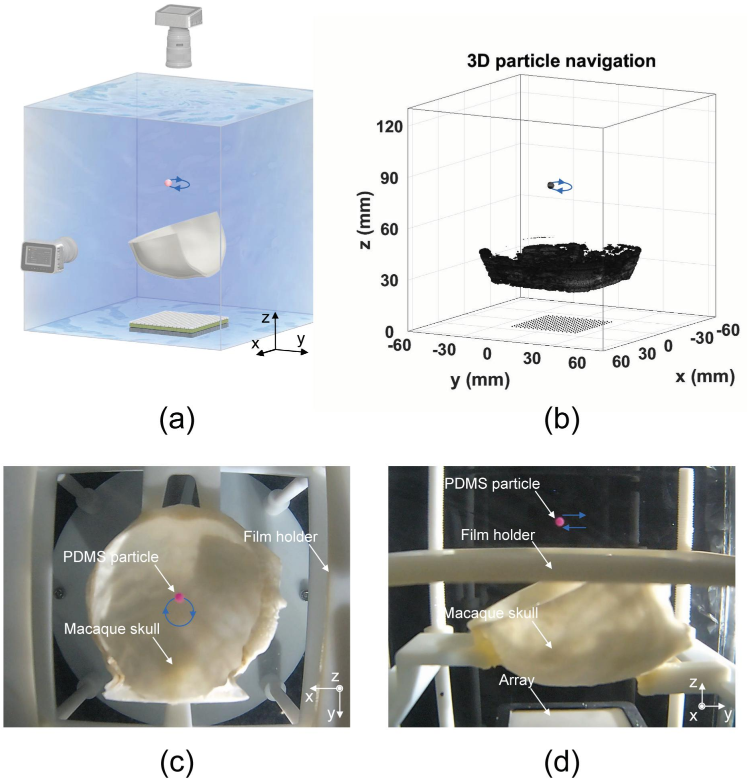

FIGURE S10. Time reversal acoustic tweezers through *ex vivo* macaque skull. (a), Schematic of the time reversal acoustic tweezers through the *ex vivo* macaque skull, which manipulates the PDMS particle in a circular path (diameter = 12 mm). (b), The 3D ultrasonic image of manipulated particle and schematic of the motion trajectory. (c-d), Optical images of the manipulated particle (dyed red) through the macaque skull and schematic of the motion trajectory in top view (c) and side view (d). The time-reversal acoustic tweezers through the *ex vivo* macaque skull are shown in Movie S3. All scale bars are 10 mm.
